# Supplementary material for: Risk factors and outcome due to extended-spectrum β-lactamase-producing uropathogenic Escherichia coli in community-onset bloodstream infections: A ten-year cohort study in Sweden
Source: PLoS One. 2022 Nov 3;17(11):e0277054. doi: 10.1371/journal.pone.0277054 (PMC9632835; doi:10.1371/journal.pone.0277054)
Supplement: S1 Table — ESBL UPEC vs. Non-ESBL UPEC. (DOCX) [file pone.0277054.s001.docx]

**S1 Table.** Laboratory data in Emergency Department. ESBL UPEC vs. Non-ESBL UPEC

|  | | ESBL UPEC  (n=77) | Non-ESBL UPEC  (n=77) | p-value |
| --- | --- | --- | --- | --- |
| Laboratory on admission (25^th^-75^th^ percentiles) | |  |  |  |
|  | C-reactive protein (mg/L) | 88 (22-218), n=76 | 179 (70-232), n=76 | 0.055 |
|  | White blood cell count (x10^9^/L) | 12 (8-17), n=77 | 12 (10-18), n=77 | 0.953 |
|  | Platelet count (x10^9^/L) | 196 (146-256), n=77 | 201 (160-262), n=77 | 0.312 |
|  | Creatinine (µmol/L) | 97 (80-143), n=72 | 96 (78-128), n=74 | 0.584 |
|  | eGFR MDRD (mL/min/1.73 m^2^) | 60 (41-77), n=49 | 59 (41-82), n=56 | 0.777 |
|  | Albumin (g/L) | 32 (29-37), n=35 | 31 (26-34), n=38 | 0.086 |
|  | Haemoglobin (g/L) | 136 (121-147), n=77 | 127 (117-137), n=77 | 0.157 |
|  | Procalcitonin (µg/L) | 3 (0.3-24), n=14 | 3 (0.6-33), n=10 | 0.613 |
|  | Lactate (µmol/L) | 1.8 (1.3-3.5), n=25 | 2 (1.3-3), n=20 | 0.506 |
| Vital sign on admission (25^th^-75^th^ percentiles) | |  |  |  |
|  | Body temp (BT) | 38.7 (38.1-39.7), n=77 | 38.3 (37.6-39.1), n=74 | 0.006 |
|  | Systolic blood pressure (SBP) | 137 (120-154), n=73 | 134 (121-150), n=74 | 0.532 |
|  | Diastolic blood pressure (DBP) | 76 (65-87), n=64 | 76 (65-86), n=66 | 0.696 |
|  | Pulse rate (PR) | 98 (86-110), n=75 | 99 (85-115), n=75 | 0.912 |
|  | Respiratory rate (RR) | 20 (18-28), n=72 | 21 (16-26), n=70 | 0.103 |
|  | Blood O_2_ saturation (SPO_2_) | 95 (91-98), n=75 | 96 (92-98), n=73 | 0.972 |
|  | Reaction Level Scale (RLS) | 1 (1-1), n=75 | 1 (1-1), n=74 | 0.809 |

*Data are presented as median, interquartile 25^th^ to 75^th^ percentile range and Mann–Whitney U. P values < 0.05 are shown in italics.*
