# Supplementary material for: Risk factors and outcome due to extended-spectrum β-lactamase-producing uropathogenic Escherichia coli in community-onset bloodstream infections: A ten-year cohort study in Sweden
Source: PLoS One. 2022 Nov 3;17(11):e0277054. doi: 10.1371/journal.pone.0277054 (PMC9632835; doi:10.1371/journal.pone.0277054)
Supplement: S2 Table — (DOCX) [file pone.0277054.s002.docx]

**S2 Table.** Distribution of ST-type ESBL UPEC vs. non-ESBL UPEC

| MLST | ESBL UPEC n=77 (%) | Non-ESBL UPEC n=80 (%) |
| --- | --- | --- |
| ST131 | 42 (54.5) | 4 (5) |
| ST69 | 1 (1.3) | 12 (15) |
| ST73 | 0 | 11 (13.8) |
| ST95 | 0 | 10 (12.5) |
| ST38 | 8 (10.4) | 1 (1.3) |
| ST405 | 7 (9.1) | 0 |
| Non-conclusive | 0 | 7 (8.8) |
| ST127 | 0 | 5 (6.3) |
| ST14 | 2 (2.6) | 1 (1.3) |
| ST12 | 2 (2.6) | 0 |
| ST162 | 2 (2.6) | 0 |
| ST58 | 0 | 3 (3.8) |
| ST130 | 0 | 2 (2.5) |
| ST404 | 0 | 2 (2.5) |
| ST648 | 2 (2.6) | 0 |
| Others STs | 11 (14.3) | 22 (27.5) |

*Distribution of ST types. The following ST types were present among cases but only in one isolate: ST 23, ST 48, ST 117, ST 617, ST 648, ST 746, ST 973, ST 1193, ST 1284, ST 2025, ST 8131.*

*The following ST types were present among controls but only in one isolate: ST 10, ST 59, ST 62, ST 101, ST 117, ST 135, ST 141, ST 349, ST 357, ST 362, ST 393, ST 420, ST 676, ST 1139, ST 1236, ST 2015, ST 2792, ST4121, ST 6316, ST 8186, ST 8541.*
